# Real life clinical outcomes of relapsed/refractory diffuse large B cell lymphoma in the rituximab era: The STRIDER study

**DOI:** 10.1002/cam4.7448

**Published:** 2024-07-19

**Authors:** Irene Dogliotti, Veronica Peri, Michele Clerico, Francesco Vassallo, Davide Musto, Silvio Mercadante, Simone Ragaini, Barbara Botto, Mario Levis, Mattia Novo, Marco Ghislieri, Luca Molinaro, Umberto Mortara, Chiara Consoli, Alessio Lonardo, Giulia Bondielli, Simone Ferrero, Roberto Freilone, Umberto Ricardi, Benedetto Bruno, Federica Cavallo

**Affiliations:** ^1^ Division of Hematology University Hospital A.O.U. “Città della Salute e della Scienza” Turin Italy; ^2^ Division of Hematology U, Department of Molecular Biotechnologies and Health Sciences University of Turin Turin Italy; ^3^ Division of Hematology Ospedale Santa Croce e Carle Cuneo Italy; ^4^ Division of Hematology AOU “Città della Salute e della Scienza di Torino” Turin Italy; ^5^ Division of Radiotherapy, Department of Oncology. University Hospital A.O.U. “Città della Salute e della Scienza” Turin Italy; ^6^ Department of Electronics and Telecommunications Politecnico di Torino Turin Italy; ^7^ PolitoBIOMed Lab, Politecnico di Torino Turin Italy; ^8^ Division of Pathology AOU “Città della Salute e della Scienza di Torino” Turin Italy

**Keywords:** chemotherapy, diffuse large B cell lymphoma, real world, refractory disease, relapse, rituximab, transplantation

## Abstract

**Background:**

Relapse and refractory (R/R) rates after first‐line R‐CHOP in diffuse large B cell lymphomas (DLBCL) are ~40% and ~15% respectively.

**Aims:**

We conducted a retrospective real‐world analysis aimed at evaluating clinical outcomes of R/R DLBCL patients.

**Material and Methods:**

Overall, 403 consecutive DLBCL patients treated in two large hematological centers in Torino, Italy were reviewed.

**Results:**

At a median follow up of 50 months, 5‐year overall survival from diagnosis (OS‐1) was 66.5%, and 2‐year progression free survival (PFS‐1) was 68%. 134 (34.4%) patients relapsed (*n* = 46, 11.8%) or were refractory (*n* = 88, 22.6%) to R‐CHOP. Most employed salvage treatments included platinum salt‐based regimens in 38/134 (28.4%), lenalidomide in 14 (10.4%). Median OS and PFS after disease relapse or progression (OS‐2 and PFS‐2) were 6.7 and 5.1 months respectively. No significant difference in overall response rate, OS‐2 or PFS‐2 in patients treated with platinum‐based regimens versus other regimens was observed. By multivariate analysis, age between 60 and 80 years, germinal center B cell type cell of origin and extranodal involvement of <2 sites were associated with better OS‐2.

**Discussion:**

Our findings confirm very poor outcomes of R/R DLBCL in the rituximab era. Widespread approval by national Medicine Agencies of novel treatments such as CAR‐T cells and bispecific antibodies as second‐line is eagerly awaited to improve these outcomes.

## 1 INTRODUCTION

Clinical outcomes of patients with diffuse large B cell lymphoma (DLBCL) remain suboptimal with relapse and chemo‐refractoriness rates up to 40%–50% and 10%–15% respectively.^1^, ^2^ In the relapsed/refractory (R/R) population, disease‐specific features such as MYC and BCL2/BCL6 translocations (i.e., double or triple hit lymphoma) or protein expression (i.e., double/triple expressors), and unfavorable gene expression signatures are associated with very poor outcomes.^3^, ^4^, ^5^, ^6^, ^7^, ^8^ The “classical” first‐line treatment backbone, containing an anthracycline and an anti CD20 antibody (commonly “R‐CHOP”: rituximab, cyclophosphamide, vincristine, doxorubicin, prednisone),^9^, ^10^, ^11^ has recently been challenged by the introduction of novel agents such as first‐line polatuzumab vedotin,^12^ while salvage treatment in the R/R setting has not yet been standardized, particularly for elderly patients.^1^ Even though second‐line combinations with platinum salts and cytarabine, or etoposide, and rituximab (i.e., R‐DHAP: rituximab, cytarabine, cisplatin and dexamethasone; R‐OxDHA: rituximab, oxaliplatin, cytarabine and dexamethasone; R‐ICE: rituximab, ifosfamide, etoposide, carboplatin; R‐GDP: rituximab, gemcitabine, dexamethasone and cisplatin), followed by autologous stem cell transplantation (ASCT) as consolidation, are commonly employed for medically fit patients,^13^, ^14^, ^15^, ^16^ population‐based studies report that over half of R/R patients undergo mainly palliation.^17^ For subsequent relapses, approved agents in Italy include single agent pixantrone and lenalidomide, with a median OS ranging between 8 and 10 months.^18^, ^19^ Allogeneic stem cell transplantation (allo‐SCT) is used only in selected cases with good response to re‐induction therapy, with 4‐year overall survival (OS) around 20%; its feasibility, however, is limited by patient age, comorbidities, and risk of treatment‐related toxicities.^20^, ^21^

Recently, several novel strategies have been developed. However, their approval relies on national Medicine Agencies the policies of which differ significantly from country to country. The U.S. Food and Drug Administration (FDA) approved chimeric antigen receptor (CAR) T cell therapies axicabtagene ciloleucel (axi‐cel) and tisagenlecleucel (tisa‐cel), in 2017 and 2018, respectively, for adult patients with high‐grade B‐cell lymphomas or DLBCL R/R to at least 2 therapy lines. In the ZUMA 1 and JULIET studies, overall response rate (ORR) were 82% and 52% with a complete response (CR) rate of 54% and 40% respectively.^22^, ^23^ In the TRANSCEND study,^24^ similar results were reported with lisocabtagene maraleucel, approved in 2021, and later extended also to refractory patients and to those relapsing within 12 months after first line treatment in June, 2022, according to the results of the TRANSFORM study.^25^ In long term safety and efficacy analyses median OS at 24 months was not reached for both axi‐cel^26^ and tisa‐cel in patients who achieved CR at 3 and 6 months.^27^ Recently, axi‐cel was FDA‐ approved for DLBCL refractory to first‐line chemo‐immunotherapy based on the ZUMA‐7 study.^28^ Other recently approved agents include: tafasitamab, an Fc‐enhanced, humanized, monoclonal antibody targeting CD19, used in combination with lenalidomide in adult patients not eligible for ASCT^29^; the antibody‐drug conjugate polatuzumab vedotin, a CD79b‐directed antibody conjugated with monomethyl auristatin (MMAE), used in combination with bendamustine and rituximab after at least two prior therapies^30^; loncastuximab tesirine, a CD19‐directed antibody and alkylating agent conjugate^31^; and the XPO inhibitor selinexor used as single agent after two to five systemic treatment lines.^32^

Here, we present a study designed to determine real‐life clinical outcomes of R/R DLBCL in the rituximab era, and to possibly characterize baseline features at diagnosis that may predict poor response to first‐line treatment and response to salvage therapies.

## 2 MATERIALS AND METHODS

### 2.1 Study design

The “STRIDER” (“strategies of treatment in diffuse large B cell lymphoma in the era of rituximab”) is a retrospective, observational study designed to evaluate clinical outcomes of R/R DLBCL patients after first‐line treatment in the rituximab era in a real‐world setting. Between January 2010 and December 2019, patients older than 18 years, consecutively treated at 2 Tertiary Referral Centers (Division of Hematology—University of Torino, Italy, and Division of Hematology, AOU Città della Salute e della Scienza—Torino, Italy), were evaluated for enrollment. The study was proposed and discussed with the patients by the treating hematologist during follow up visits. Patients were enrolled after obtaining informed consent. The study was approved by the local Institutional Review Board (A.O.U. Città della Salute e della Scienza, Torino, Italy) and conducted according to the Declaration of Helsinki. Patient data were obtained from hospital health records and research files. All data were pseudo‐anonymized by assignment of a study specific patient code.

### 2.2 Inclusion criteria

Major inclusion criteria included initial biopsy‐proven diagnosis of either DLBCL or high grade B cell lymphomas (HGBCL). Histological and immune‐histochemical diagnosis by tru‐cut core‐needle biopsies was allowed, while cases diagnosed by fine needle aspiration cytology were excluded. Transformed disease after a previous diagnosis of low grade lymphoma was allowed. R/R disease was documented by biopsy, imaging studies or clinical evaluation; refractoriness to first line treatment was defined as reappearance or progression of DLBCL or HGBCL within 12 months from initial diagnosis or disease‐related death (POD‐12). For all patients, retrieved data included demographics, whole blood counts, basic metabolic panel; imaging studies (computed tomography, CT, and fluorodeoxyglucose*‐positron emission tomography*, PET, if available); histology studies by lymph node biopsy, bone marrow biopsy and bone marrow aspirate; presence of B symptoms, performance status, prognostic scores [IPI, age‐adjusted IPI, Central Nervous S,35ystem International Prognostic Index (CNS‐IPI)]; number of therapy lines and regimens employed. Cell of origin (COO) was determined by Hans' algorithm method; bulky disease was defined as any lesion >6 cm by CT.

### 2.3 Statistical analysis

Primary endpoint was OS for R/R patients after salvage treatment (OS‐2). Secondary endpoints included progression free survival after first line treatment (PFS‐1), PFS after salvage treatment defined as PFS‐2; OS‐1; POD12; determinants for survival outcomes; distribution of second line regimens (descriptive analysis); efficacy outcomes with salvage treatments (response and duration of response). Sample size estimation was not predefined, all consecutive patients meeting the inclusion criteria during the defined study period were eligible for the study though patients with missing data were excluded from the analysis. Response to therapy was determined by the 1999 International Working Group response criteria^33^ and by Lugano response criteria for patients diagnosed after 2014.^34^ Baseline characteristics of R/R patients were compared to those of non R/R (NRR) patients to identify potential prognostic factors. Statistical analyses were carried out using R (v 4.3.1). Survival curves were plotted with Kaplan–Meier method and compared with log‐rank test. Medians between groups for continuous variables were compared by the Kruskal–Wallis (for non‐normal variables) or the one‐way ANOVA test (for normal variables); the chi‐squared test or Fisher's exact test for small study samples, were employed for categorical variables. The Cox proportional hazards model was implemented for the univariate and multivariate survival analyses. In particular, an AIC‐based backward stepwise algorithm (R function stats::step) was used to perform the variable selection, from 12 initial variables (outcome after first‐line, age, hemoglobin, COO, gender, ECOG performance status, stage, extranodal involvement, LDH, ki67, type of first‐ and second‐line treatment) to 6 (age, hemoglobin, COO, extranodal involvement, ki67, type of second line treatment). This method allows to determine the most relevant covariates for the outcomes of interest and to analyze potential confounding factors for each covariate. As required by the algorithm, the dataset was restricted to the R/R patients for whom there were no missing data on the initial 12 variables (*n* = 48). Thus, multivariate analysis for OS‐2 was re‐run by applying a data imputation algorithm, the “Multiple Imputation by Chained Equations” (MICE), a robust, informative method to analyze datasets with missing data. The procedure “fills in” missing data through an iterative series of predictive models. In each iteration, a specified variable is imputed using the other variables in the dataset. These iterations are run until convergence has been met. POD12 was determined by logistic regression: the model predicts the probability of being refractory/relapsed/dead due to DLBCL within 12 months and the causing factors.

## 3 RESULTS

### 3.1 Baseline patient characteristics

**FIGURE 1:**
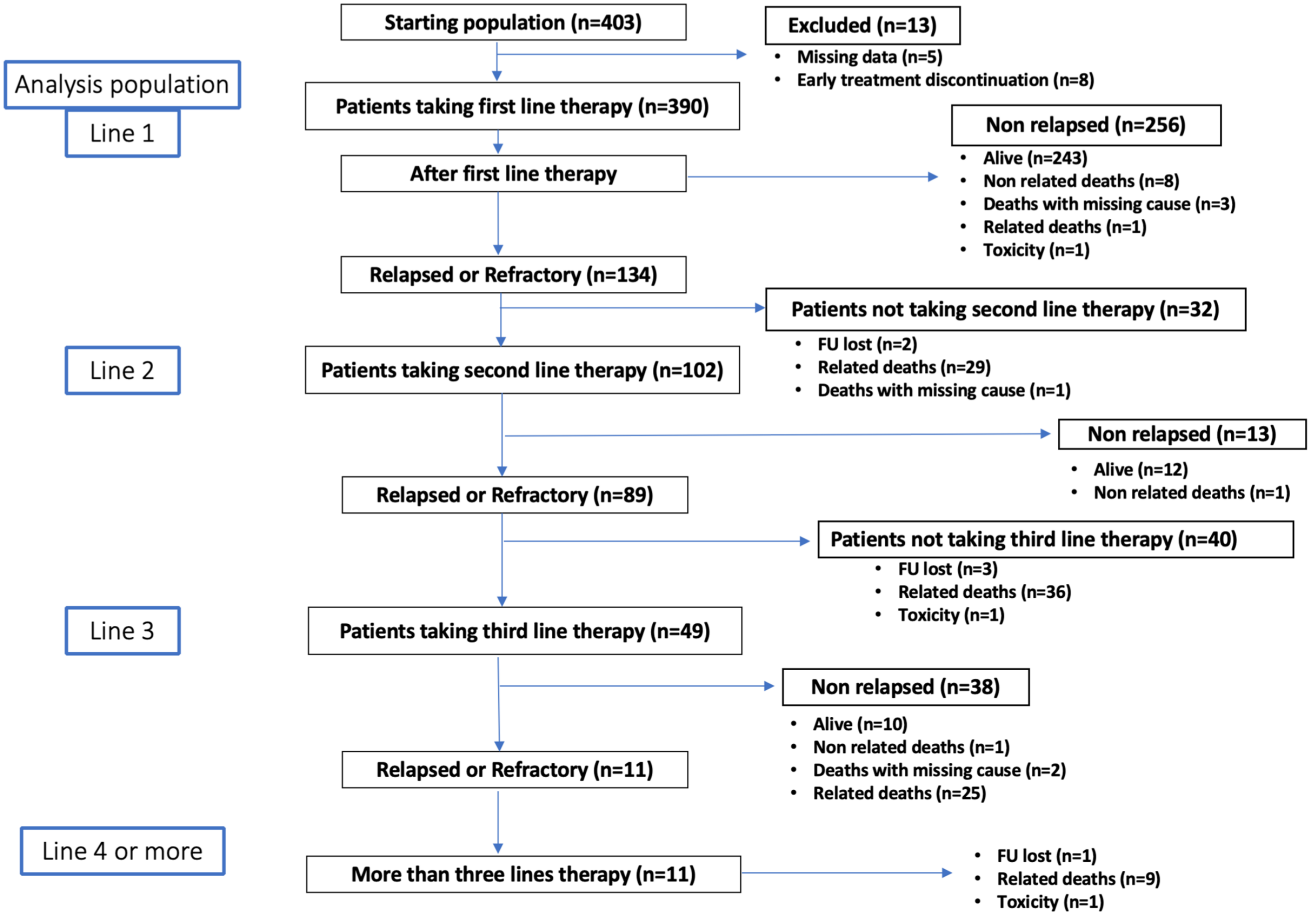
The STRIDER study: Graphical representation of enrollment, treatment, and follow‐up of 403 patients.

Four‐hundred‐three patients met the inclusion criteria, Thirteen were not included in the final analysis because of incomplete data (*n* = 8) or early treatment discontinuation (*n* = 5) (Figure 1). Among patients included in the final analyses, 384 had DLBCL, NOS, while 6 (2%) had HGBCL. At follow up, after first line therapy, 256/390 patients (65.6%) were still in first response, whereas 134 (34.4%) had either relapsed (*n* = 46, 11.8%) or were refractory (*n* = 88, 22.6%). Patient characteristics at diagnosis are shown in Table 1. Median age was similar in the “response” and in the “relapse” groups (67.7 and 66.3 years respectively), though significantly lower as compared to the “refractory” patient group (75.0 years) (*p* < 0.001). High baseline IPI score (categorized as 0–1, 2–3, 4–5) (*p* < 0.001), advanced stage (*p* < 0.001), B symptoms (*p* < 0.001), and ≥2 extra‐nodal site involvement (*p* = 0.035) were more frequent among patients with R/R disease. At diagnosis, CNS involvement was observed in 7/390 (1.8%) patients; overall, high CNS IPI risk was present in 53 (20.7%) for the NRR group, while in 12 (26.1%) and 40 (45.5%) for relapsed and refractory patients respectively (*p* < 0.001). Bulky disease was present in 71 (27.7%), 18 (39.1%) and 42 (47.7%) of NRR, relapsed and refractory patients (*p* < 0.001) respectively. The expression patterns of conventional immune‐histochemical markers (namely CD20, BCL2, BCL6, C‐MYC) in the diagnostic lymph node biopsy did not significantly differ between groups.

**TABLE 1:** Patient characteristics and treatments.

	All	NRR	R/R	
			Relapsed disease	Refractory disease
	*N* (%)	*N* (%)	*N* (%)	
Total patients	**390 (100.0)**	**256 (65.6)**	**134 (34.4)**	
			46 (11.8)	88 (22.6)
Baseline characteristics				
Sex				
Male	218 (55.9)	138 (53.9)	33 (71.7)	47 (53.4)
Female	172 (44.1)	118 (46.1)	13 (28.3)	41 (46.6)
Median age (years [IQR])	68.6 [58.8–76.7]	67.7 [56.8–74.6]	66.3 [58.7–75.5] 75.0 [63.0–81.2]	
Stage at diagnosis				
1–2	95 (24.4)	79 (30.9)	8 (17.4)	8 (9.1)
3–4	291 (74.6)	175 (68.4)	37 (80.4)	79 (89.8)
Missing	4 (1.0)	2 (0.8)	1 (2.2)	1 (1.1)
B symptoms at diagnosis	131 (33.6)	69 (27.0)	18 (39.1)	44 (50.0)
Missing	2 (0.5)	0 (0)	1 (2.2)	1 (1.1)
ECOG_PS				
0–1	318 (81.5)	210 (82.0)	38 (82.6)	70 (79.5)
2–4	65 (16.7)	44 (17.2)	6 (13.0)	15 (17.0)
Missing	7 (1.8)	2 (0.8)	2 (4.3)	3 (3.4)
Extranodal sites involvement (≥2 sites)	173 (44.4)	102 (39.8)	23 (50.0)	48 (54.5)
Missing	4 (1.0)	2 (0.8)	1 (2.2)	1 (1.1)
IPI Score				
0–1	81 (20.8)	70 (27.3)	6 (13.0)	5 (5.7)
2–3	201 (51.5)	132 (51.6)	26 (56.5)	43 (48.9)
4–5	102 (26.2)	51 (19.9)	12 (26.1)	39 (44.3)
Missing	6 (1.5)	3 (1.2)	2 (4.3)	1 (1.1)
Bulky disease				
Yes	131 (33.6)	71 (27.7)	18 (39.1)	42 (47.7)
Missing	2 (0.5)	0 (0)	1 (2.2)	1 (1.1)
CNS‐IPI score high risk (≥4–5‐6)	105 (26.9)	53 (20.7)	12 (26.1)	40 (45.5)
Treatment intent				
Curative	374 (95.9)	251 (98.0)	43 (93.5)	80 (90.9)
Palliative	13 (3.3)	4 (1.6)	2 (4.3)	7 (8.0)
Missing	3 (0.8)	1 (0.4)	1 (2.2)	1 (1.1)
First line treatment				
R‐CHOP/R‐COMP	315 (80.8)	224 (87.5)	38 (82.6)	53 (60.2)
R‐mini CHOP/COMP	28 (7.2)	12 (4.7)	2 (4.3)	14 (15.9)
R‐CODOX‐M/R‐IVAC	9 (2.3)	4 (1.6)	1 (2.2)	4 (4.5)
DA‐EPOCH	6 (1.5)	3 (1.2)	1 (2.2)	2 (2.3)
Oral chemotherapy ± rituximab	24 (6.2)	11 (4.3)	3 (6.5)	10 (11.4)
Missing	8 (2.1)	2 (0.8)	1 (2.2)	5 (5.7)
Second line treatment	**102 (100.0)**			
Platinum based^a^	38 (37.3)	‐	16 (39.0)	22 (36.1)
Lenalidomide	14 (13.7)	‐	5 (12.2)	9 (14.8)
Clinical trial	2 (2.0)	‐	2 (4.9)	0 (0.0)
Other	37 (36.3)	‐	15 (36.6)	22 (36.1)
Oral chemotherapy ± rituximab	11 (10.8)	‐	3 (7.3)	8 (13.1)
Second line consolidation				
RT	11 (10.8)	‐	6 (14.6)	5 (8.2)
ASCT	6 (5.9)	‐	5 (12.2)	1 (1.6)
Missing	49 (48.0)		19 (46.3)	30 (49.2)

Abbreviations: ASCT, autologous stem cell transplant; CNS, central nervous system; ECOG_PS, Eastern Cooperative Oncology Group_performance status; IPI, international prognostic index; NRR, not relapsed and not refractory disease; R/R, relapsed or refractory disease; R‐CHOP/COMP, Rituximab, doxorubicin, vincristine, cyclophosphamide and prednisone (with liposomal doxorubicin in case of previous cardiovascular disease); R‐CODOX‐M, rituximab, cyclophosphamide, vincristine, doxorubicin and methotrexate; R‐DA EPOCH, rituximab, etoposide, vincristine, cyclophosphamide and prednisone; R‐IVAC, rituximab, ifosfamide, etoposide, cytarabine; RT, radiotherapy.

The significance is *p* < 0.05 are in bold.

^a^ Platinum‐based treatments: rituximab + OxDHA or DHAP or GDP or GemOx.

### 3.2 First‐line treatments

Most patients (374/390, 95.9%) underwent chemotherapy with a curative intent (Table 1): 315 (80.8%) were treated with standard R‐CHOP/COMP (liposomal doxorubicin in case of previous cardiovascular disease), 28 (7.2%) with R‐mini‐CHOP/COMP, 9 (2.3%) underwent alternating R‐CODOX‐M (rituximab, cyclophosphamide, vincristine, doxorubicin and methotrexate) and R‐IVAC (rituximab, ifosfamide, etoposide, cytarabine), 6 (1.5%) R‐DA EPOCH (rituximab, etoposide, vincristine, cyclophosphamide and prednisone), 24 (6.2%) were treated with oral chemotherapy ± rituximab. CNS prophylaxis with intrathecal methotrexate was used in 73 patients (18.7%), while i.v. methotrexate in 20 (5.1%). Following first‐line therapy, 101/390 (25.9%) patients received consolidation radiotherapy (30–36 Gy) on bulky disease, while 4 (1.0%) received (ASCT).

### 3.3 Second‐ and third‐line treatments

Overall, 102/134 patients received second‐line treatments. Salvage therapies included high dose, platinum‐based (rituximab + OxDHA/DHAP/GemOx) regimens in 38/102 (37.3%) and 6 (5.9%) underwent ASCT. Of note, all patients that were deemed eligible for ASCT in terms of age (<70 years for standard dose conditioning with BEAM/FEAM schemes, <75 for reduced intensity conditioning) and comorbidities received high dose treatments. Other treatments included lenalidomide (*n* = 14, 13.7%), investigational drugs in clinical trials (*n* = 2, 2.0%), and miscellaneous regimens not containing platinum, mainly oral chemotherapy ± rituximab. Median age was 62 years for patients who received platinum‐based regimens versus 75.5 years for those who did not (*p* < 0.001). Second‐line consolidation with radiotherapy was performed in 11 (10.8%) patients (Table 1). Forty‐nine patients R/R to second‐line therapies received third‐line treatments, including lenalidomide (*n* = 13/49, 26.5%), platinum‐based regimens (*n* = 2, 4.0%), investigational drugs (*n* = 1, 2.0%) and others (*n* = 33, 67.3%). For patients who did not receive any second‐line treatment (*n* = 32), main reason was rapidly progressive disease, with a median time from progression to disease‐related death of 15 days. Median age of this subset of patients was 79.4 years old, at initial diagnosis 10 patients (31.3%) had ECOG PS 2–4, and 14 patients (43.8%) had IPI score 4–5; 23 patients (71.9%) had elevated LDH.

### 3.4 Response to treatments

After first‐line, ORR was 81.0% (316/390) including 72.6% complete remission (CR) and 8.5% partial remission (PR); 4.6% of patients had incomplete response data. Overall, only 102 (76.1%) of 134 R/R patients underwent second‐line therapy (Figure 1). Reasons for not receiving salvage treatment included disease related death in 29/32 (90.6%) patients. After second‐line, ORR was 59.8% (61/102), with CR in 48% patients, PR in 11.8%, stable disease in 2.9% and progressive disease in 34.3% patients; in 2.9% patients data were incomplete. Eighty‐nine patients (87.3% of 102 who underwent second‐line therapy) were R/R to second‐line treatment. ORR after second‐line platinum based chemotherapy was 60.5%; in particular 18/38 (48.6%) patients achieved CR and 5 (13.5%) PR; ORR with other regimens was similar (59.4% with 50% CR). Outcomes with second‐line lenalidomide included 64.3% ORR with 50.0% patients achieving CR, though response duration was short. Third‐line therapy was feasible in 49/89 (55.1%), while the remaining rapidly deceased due to DLBCL progression (*n* = 36/40, 90.0%, Figure 1). Among those who received third‐line therapy, only 10/49 (20.4%) were alive at last follow up.

### 3.5 Survival outcomes

**FIGURE 2:**
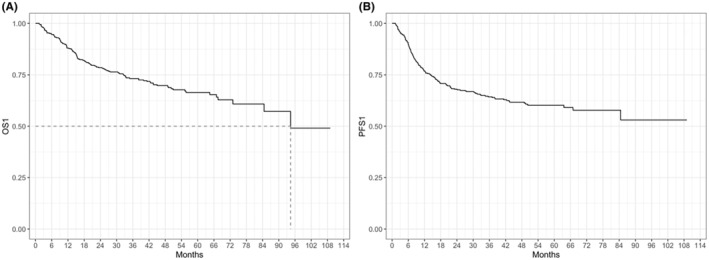
Overall survival (OS1, A) and progression free survival (PFS1, B) from diagnosis (all patients).

**FIGURE 3:**
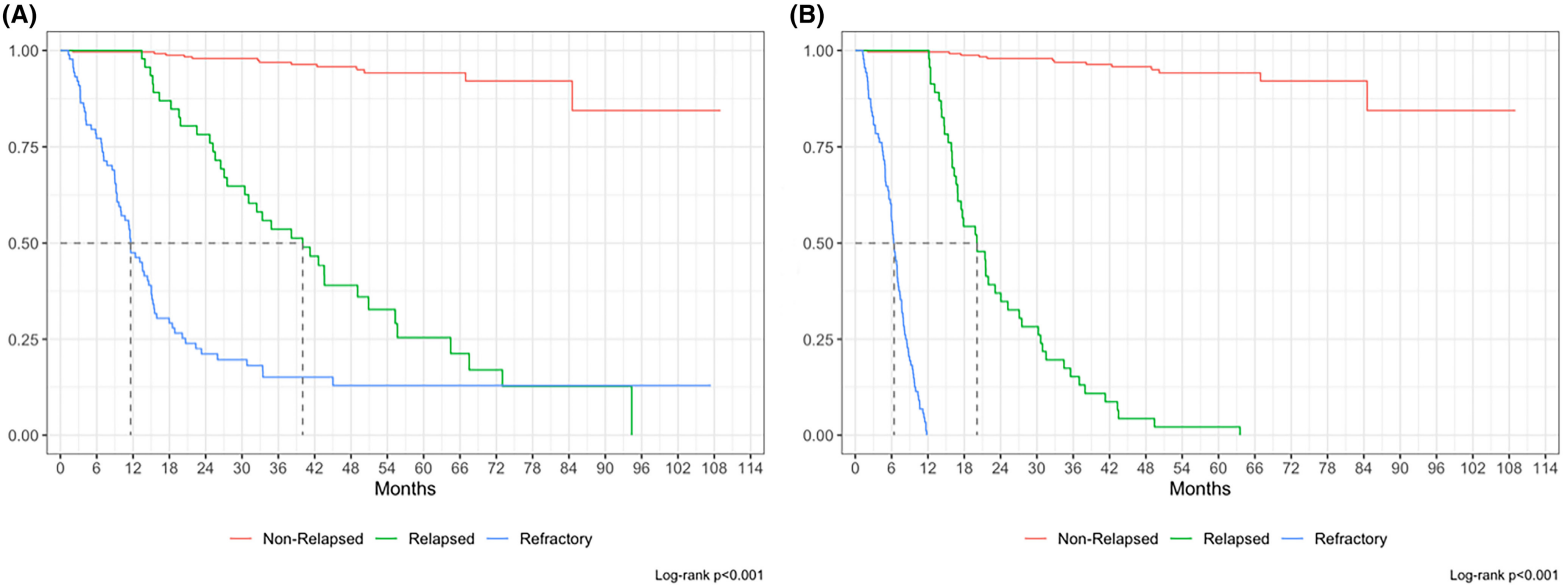
Overall survival (OS1, A) and progression free survival (PFS1, B) for non‐relapsed/refractory patients, relapsed and refractory patients.

**FIGURE 4:**
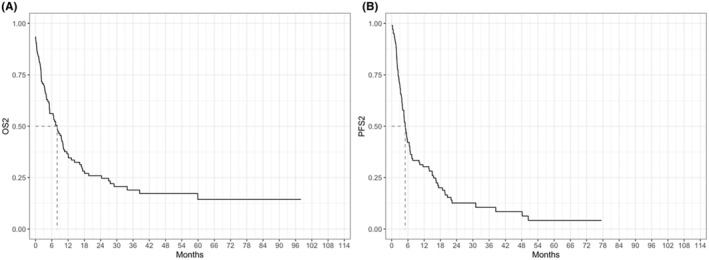
Overall survival after first relapse/progression (OS2, A) and progression free survival after first relapse/progression (PFS2, B) from diagnosis.

**FIGURE 5:**
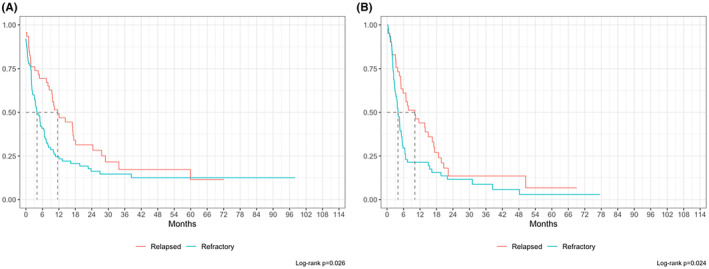
Overall survival (OS2, A) and progression free survival (PFS2, B) from second line treatment for relapsed and refractory patients by univariate analysis.

At a median follow up of 50 months, overall 5‐year OS from diagnosis (OS‐1) was 66.5% (Figure 2A). However, median OS‐1 for NRR patients was not reached with 243/256 patients (94.9%) alive at last follow up, whereas median OS‐1 was 40.0 months (IQR 25.2–64.5) for relapsed and 11.6 months (IQR 6.8–20.7) for refractory patients respectively (Figure 3A). Overall median PFS‐1 was not reached with a 2‐year PFS‐1 of 68.8% (CI 63.5%–72.9%, Figure 2B), whereas median PFS‐1 was 20.0 months (15.9–30.6) for relapsed and 6.4 months (IQR 4.5–8.5) for refractory patients respectively (Figure 3B). Overall median OS‐2 and PFS‐2 were 6.7 (IQR 1.8–18.1), and 5.1 (IQR 2.4–15.5) months respectively (Figure 4). OS‐2 was 11.6 months (IQR 3.2–29.0) for relapsed and 4 months (IQR 1.8–10.7) for refractory patients (Figure 5A), while median PFS‐2 was 10.1 months (IQR 3.8–18.8) and 4 months (IQR 2.2–6.7) respectively (Figure 5B). In R/R patients (*n* = 107) treated with first‐line R‐CHOP‐like (including R‐CHOP/COMP and mini‐RCHOP/COMP), OS‐2 and PFS‐2 did not significantly differ (median OS‐2: 8, median PFS‐2: 5.2 months).

### 3.6 Prognostic factors for survival

**FIGURE 6:**
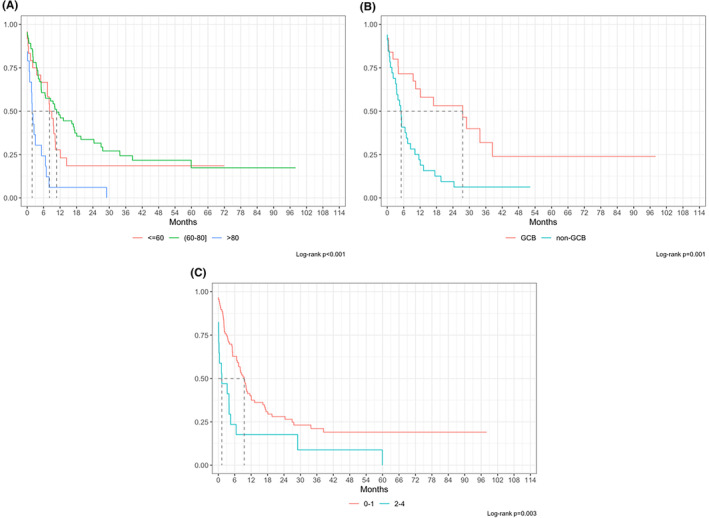
Overall survival (OS2) stratified by age (A), cell of origin (COO) (B) and by ECOG PS (C) at initial diagnosis by univariate analysis.

**FIGURE 7:**
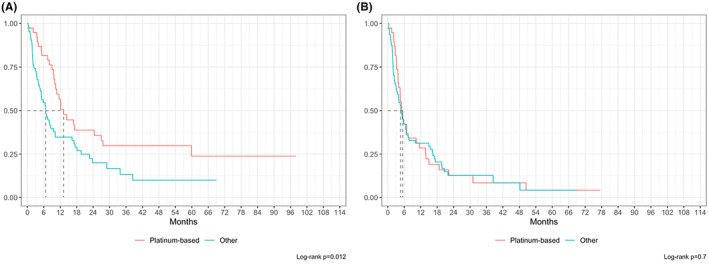
Overall survival (OS2, A) and progression free survival (PFS2, B) with platinum‐based versus other regimens as second line treatment by univariate analysis.

Among 134 R/R patients, factors significantly associated with OS‐2, by univariate analysis, were disease relapse (*p* = 0.027, HR = 1.6), age older than 80 years (*p* = 0.002, HR = 2.4), non‐GCB COO (*p* = 0.004, HR = 2.2), male gender (*p* = 0.011, HR = 0.6), ECOG PS 2–4 (*p* = 0.014, HR = 1.9), high‐dose first‐line treatment (*p* = 0.011, HR = 2.5), first‐line palliative treatment (*p* = 0.011, HR = 2.4), and salvage treatment other than platinum‐based regimens (*p* = 0.014, HR = 1.8) (Table 2). After adjusting for confounders, significant variables for OS‐2 were age between 60 and 80 years (*p* = 0.009, HR = 0.20), non‐GCB COO (*p* = 0.010, HR = 2.89), ECOG PS 2–4 (*p* = 0.016, HR = 2.34), extranodal involvement ≥2 sites (*p* = 0.026, HR = 2.34) (Figure 6). Similarly, significant variables for PFS‐2 were first‐line outcome (*p* = 0.024), extranodal involvement (*p* = 0.031), LDH (*p* = 0.017); after adjusting for confounders in a no‐missing dataset (*n* = 55), no variable was statistically significant (*p* > 0.05, Table 2). In particular, there was no statistically significant difference in PFS‐2 or OS‐2 between patients who received platinum‐based regimens as compared to those who received other therapies (Figure 7).

**TABLE 2:** Univariate analysis for OS2 and PFS2 by Cox proportional hazards model.

	OS2		*p*‐Value	PFS2		*p*‐Value
Characteristics	HR	95% CI		HR	95% CI	
Outcome						
Relapsed						
Refractory	1.6	1.0–2.3	**0.027**	1.6	1.0–2.5	**0.025**
Age class						
<60 years						
60–80 years	0.8	0.4–1.3	0.349	0.7	0.4–1.2	0.170
>80 years	2.4	1.4–4.2	**0.002**	1.513	0.8–2.9	0.210
Sex						
Female						
Male	0.6	0.4–0.9	**0.011**	0.8	0.5–1.2	0.345
Hb						
≤10 g/dL						
>10 g/dL	0.9	0.6–1.5	0.797	1.8	0.9–3.5	0.095
COO Hans						
GCB						
Non GCB	2.2	1.3–3.9	**0.004**	1.4	0.8–2.5	0.220
ECOG						
0–1						
2–4	1.8	1.1–3.0	**0.014**	0.9	0.4–1.7	0.666
Stage						
1–2						
3–4	1.0	0.6–1.9	0.788	1.2	0.6–2.1	0.567
Extranodal disease						
0–1						
≥2	1.2	0.8–1.7	0.442	1.6	1.0–2.4	**0.032**
Ki‐67						
≤70%						
>70%	1.4	0.7–2.2	0.112	1.3	0.8–2.0	0.344
LDH						
0						
1	1.4	0.9–2.3	0.126	1.9	1.1–3.2	**0.019**
Type of treatment						
Anthracycline						
High doses	2.5	1.2–5.1	**0.011**	2.2	1.0–4.8	**0.054**
Palliative	2.4	1.2–4.6	**0.011**	1.8	0.8–3.9	0.133
Salvage treatment						
Platinum based						
Other	1.8	1.1–2.9	**0.014**	1.0	0.7–1.6	0.744

Abbreviations: COO, cell of origin; ECOG_PS, Eastern Cooperative Oncology Performance Status; GCB, germinal centre B‐cell; Hb, hemoglobin; HR, hazard ratio; LDH, lactate dehydrogenase; OS2, overall survival from relapse; PFS2, progression free survival from relapse.

The significance is *p* < 0.05 are in bold.

By univariate analysis, OS‐1 was significantly associated with disease relapse (*p* < 0.0001, HR for relapsed: 26.7, for refractory: 66.2), age (*p* < 0.0001, HR for patients >80 years old: 5.9), Ann Arbor stage (*p* = 0.011, HR for stage III‐IV: 2.0), LDH (*p* = 0.001, HR 2.1), type of first‐line treatment (*p* = 0.006, HR for patients receiving intensive regimens: 2.7, Table 1S), while PFS‐1 was significantly associated with age (*p* < 0.0001, HR for patients >80 years old: 4.8), hemoglobin levels (*p* = 0.031, HR for patients with hemoglobin >10 g/dL: 0.61), stage (*p* = 0.001, HR for stage III‐IV: 2.2), LDH (*p* = 0.001, HR 2.0), type of first‐line treatment (*p* = 0.045, HR for patients receiving intensive regimens: 2.0, Table S1).

By multivariate analysis, in 48 R/R patients with a complete dataset, among 6 variables (age, hemoglobin, COO, extranodal involvement, ki67, type of second‐line treatment), age between 60 and 80 years, GCB‐type COO and extranodal involvement of <2 sites were significantly associated with OS‐2 (Table 3). By implementing the multiple imputation model for missing data of the 12 variables (*n* = 134), results did not significantly differ with age (*p* = 0.014), COO (*p* = 0.016) and number of extranodal sites (*p* = 0.034) remaining significantly associated with OS‐2. No baseline feature resulted significantly associated with PFS‐2 by multivariate analysis on 55 patients with complete data (Table 3). Moreover, logistic regression was performed to highlight determinant features for early (<12 months) R/R disease (POD12), and significant factors were age older than 80 years (*p* = 0.005, OR 8.7) and stage III‐IV (*p* = 0.011, OR 7.7, Table S2).

**TABLE 3:** Multivariate analysis for OS2 and PFS2 by Cox proportional hazards model.

Characteristics	OS2		*p*‐Value	PFS2		*p*‐Value
	HR	95% CI		HR	95% CI	
Age class						
<60 years						
60–80 years	0.2	0.1–0.7	**0.009**	0.4	0.9–1.0	0.052
>80 years	0.5	0.1–2.2	0.4	1.5	0.5–4.3	0.464
Hb						
≤10 g/dL						
>10 g/dL	4.9	0.6–40.0	0.135			
COO Hans						
GCB						
Non GCB	2.8	1.3–6.1	**0.010**	1.6	0.9–2.9	0.134
Extranodal disease						
0–1						
≥2	2.3	1.1, 4.9	**0.026**			
Ki67%						
≤70%						
>70%	1.7	0.8–3.6	0.167			
Salvage treatment						
Platinum based						
Other	2.0	0.8–5.1	0.113	1.7	0.9–3.3	0.104
Sex						
Female						
Male				1.6	0.8–3.1	0.148

Abbreviations: COO, cell of origin; ECOG_PS, Eastern Cooperative Oncology Performance Status; GCB, germinal centre B‐cell; Hb, hemoglobin; HR, hazard ratio; LDH, lactate dehydrogenase; OS2, overall survival from relapse; PFS2, progression free survival from relapse.

The significance is *p* < 0.05 are in bold.

## 4 DISCUSSION

Despite the significant improvement with the introduction of rituximab, clinical outcomes of patients with R/R DLBCL remain invariably poor. Recently, novel immunotherapies such as CAR T cells, antibody‐drug conjugates (i.e., polatuzumab vedotin, loncastuximab tesirine), and bispecific antibodies (i.e., glofitamab, epcoritamab) have however shown impressive results. Nonetheless, these agents have not yet been largely approved as second‐line therapies by many national Medicine Agencies. Thus, effective salvage treatments remain an urgent medical need. The STRIDER study is a large, retrospective, real life study that confirmed the dismal prognosis of R/R patients in the rituximab era. OS‐2 was disappointingly short with rates of about 6 and 4 months in relapsed and refractory patients respectively, with no statistically significant differences between the 2 cohorts. The SCHOLAR‐1, the largest pooled analysis on R/R DLBCL patients in the rituximab era, reported a median OS for refractory patients of 6.3 months from the start of salvage treatment.^1^

Salvage treatments commonly include platinum‐based regimens such as R‐ICE, R‐DHAP, R‐GDP followed by ASCT consolidation.^13^, ^14^, ^15^, ^16^, ^35^ Studies with DHAP and GDP reported 4‐year OS of 39%,^35^ and, with R‐DHAP and R‐ICE, 3‐year OS of 49%.^14^ Though comparative studies were not designed, ORRs did not differ significantly ranging from 40% to 60%. R‐GemOx, investigated in an older population with median age of 69 years, did not include consolidation with ASCT,^36^ reporting 5‐year OS of 14%. In our rituximab‐exposed population, ORR with platinum‐based regimens were similar to previous reports, though the initial relatively good ORR and CR rates did not translate into a survival advantage, likely due to short response duration. For this reason, many patients undergoing platinum‐based regimens did not receive ASCT consolidation, thus explaining the similar results observed with other less intensive second‐line salvage treatments. Of note, only 38/102 (37.3%) patients were however treated with platinum‐containing regimens, showing that, in a real life setting, the administration of intensive salvage treatments is not feasible in most patients. Moreover, frailty and chemo‐refractoriness, in our experience, were also documented by the fact that only 6 (15.8% of the candidates to transplant patients) receiving platinum‐based schemes eventually underwent ASCT. In published reports, the “intent‐to‐salvage transplant” was as low as 30%.^14^, ^35^

Reliable clinical parameters predicting increased risk of refractoriness prior to R‐CHOP are lacking. Given that every treatment cycle may reduce chemo‐sensitivity, it would be important to identify factors that predict survival and response to salvage treatments. Previous studies showed that high‐intermediate IPI score at relapse^37^ and secondary aaIPI^38^ affected response rate after salvage therapy while factors such as COO by Hans' algorithm at relapse,^39^ relapse <12 months after initial therapy^40^ and prior rituximab treatment^41^ also affected prognosis. We tried to identify parameters, at diagnosis, that could predict inferior outcomes in case of relapse or progression. By multivariate analysis, age category, COO, and number of extranodal sites involved were predictive of OS‐2. However, the current role of patient age should be re‐assessed in the light of novel treatments with better toxicity profile compared to standard chemotherapy. Interestingly, our patients between 60 and 80 years showed a significant survival advantage over younger patients. This could partly be explained by more aggressive biologic features in younger patients that may be detected with novel molecular signatures. Of note, a prospective study by the Fondazione Italiana Linfomi (FIL) identified age over 80 years as an independent variable correlated with OS. Moreover, a novel prognostic score for elderly patients, the EPI score, by a simplified version of the geriatric assessment (sGA) was proposed. By classifying patients as fit, unfit, and frail, the EPI score risk correlated with OS of 75%, 58%, and 43%, respectively.^42^ As a matter of fact, at our centre, a thorough geriatric assessment is part of the initial clinical work up by which elderly patients are offered treatment based on their fitness. This may be the reason for the low rates of palliative care undergone by our patients at diagnosis (3.3%) and at relapse/refractoriness (20%) compared to other reports.^17^ Indeed, patients over 80 are frequently ineligible for chemotherapy due to comorbidities.^43^ Lenalidomide may be a valid option, especially in non‐GCB patients, with reported ORRs ranging from 29% to 37% with up to 20% CR rates.^44^, ^45^ We report ORR and CR of 64.3% and 50%, respectively, in 14 patients, median age 76 years, treated with second‐line lenalidomide, whereas third‐line lenalidomide in 13 patients showed inferior outcomes. Finally, a recent real world experience reported outcomes and costs associated with CAR‐T cell therapy in DLBCL patients older than 65 (of note; however, regulatory approval in Italy actually limit access to CAR‐T programs only to patients <75 years old). Overall, median OS was 17.1 months with no difference between age groups (65–69; 70–74; >75 years).^46^ No patient in the STRIDER was treated with CAR‐T cells as the study was conducted before CAR‐T cell therapies were commercially approved after two treatment lines.

Splitting the COO category in GCB and non‐GCB (NOS) patients by immunohistochemical analysis,^47^, ^48^, ^49^ according to Hans' algorithm, evaluated at baseline in over half of our patients, identified a strong variable associated with both OS‐1 and OS‐2 by multivariate analysis. Overall, the role of COO, assessed even with more precise tools such as Nanostring platforms, has recently been questioned in favor of more complex gene signature classifications, able to detect DLBCL molecular heterogeneity and to predict clinical outcome. However, these technologies are not routinely available at most centers and are primarily used in the context of clinical trials.^5^, ^6^, ^7^, ^8^

With the emergence of new therapies, and the increasing biological understanding of DLBCL pathogenesis, efforts to re‐design first‐line treatments beyond R‐CHOP are being made. A stringent baseline risk stratification and the availability of a dynamic risk evaluation in the follow up that includes first‐line response and disease kinetics (i.e., early vs. late relapse) should allow to promptly identify poor prognosis patients, who may benefit from earlier interventions with novel immunotherapies, including CAR‐T cells.

## 5 CONCLUSIONS

The STRIDER is a large, retrospective, real life study that confirms the poor prognosis of R/R DLBCL patients in the rituximab era, before the implementation of “next generation” salvage treatments such as CAR‐T cells and bispecific antibodies. Efficacy of high dose chemotherapy‐based salvage treatments is limited, requiring the urgent and widespread approval of these novel immunotherapies, mainly investigated in clinical trials in Europe.

## AUTHOR CONTRIBUTIONS

**Irene Dogliotti:** Conceptualization (lead); data curation (equal); investigation (lead); writing – original draft (lead); writing – review and editing (equal). **Veronica Peri:** Data curation (lead); writing – original draft (equal); writing – review and editing (lead). **Michele Clerico:** Data curation (equal); writing – review and editing (equal). **Francesco Vassallo:** Data curation (equal). **Davide Musto:** Methodology (equal); software (equal). **Silvio Mercadante:** Methodology (equal); software (lead). **Simone Ragaini:** Data curation (equal); writing – review and editing (equal). **Barbara Botto:** Supervision (equal); writing – review and editing (equal). **Mario Levis:** Data curation (equal); writing – review and editing (equal). **Mattia Novo:** Data curation (equal); writing – review and editing (equal). **Marco Ghislieri:** Methodology (equal); software (equal). **Luca Molinaro:** Data curation (equal); investigation (equal). **Umberto Mortara:** Data curation (supporting). **Chiara Consoli:** Data curation (supporting); writing – review and editing (supporting). **Alessio Lonardo:** Project administration (equal). **Giulia Bondielli:** Project administration (equal). **Simone Ferrero:** Investigation (equal); resources (equal); writing – review and editing (equal). **Roberto Freilone:** Supervision (equal); writing – review and editing (equal). **Umberto Ricardi:** Supervision (equal); writing – review and editing (equal). **Benedetto Bruno:** Methodology (equal); supervision (lead); writing – original draft (equal). **Federica Cavallo:** Conceptualization (lead); funding acquisition (lead); methodology (equal); resources (equal); supervision (equal); writing – original draft (equal); writing – review and editing (lead).

## CONFLICT OF INTEREST STATEMENT

BB has received speaker honoraria from Amgen, Janssen, Novartis, BeiGene, Bristol Myers Squibb, GlaxoSmithKline, Jazz Pharmaceuticals, Astrazeneca, Incyte; has served on advisory boards for Amgen, Jazz Pharmaceuticals. SF has received research funding from Janssen, Morphosys, Gilead, Beigene; has served on advisory boards for EusaPharma, Janssen, Clinigen, Incyte, Italfarmaco, Roche, Abbvie, has served as consultant for EusaPharma, Janssen, Sandoz, Abbvie, has received speaker honoraria from Janssen, EusaPharma, Servier, Gentili, Lilly, Beigene, Gilead. FC has received speaker honoraria from Lilly, Incyte; has served on advisory boards for Roche, Incyte; has served as consultant for Astrazeneca.

## Supporting information


Table S1.

## Data Availability

The data that support the findings of this study are available from the corresponding author upon reasonable request.
