# Supplementary material for: Real life clinical outcomes of relapsed/refractory diffuse large B cell lymphoma in the rituximab era: The STRIDER study
Source: Cancer Med. 2024 Jul 19;13(14):e7448. doi: 10.1002/cam4.7448 (PMC11258436; doi:10.1002/cam4.7448)
Supplement: Supplementary file 1 — Table S1. [file CAM4-13-e7448-s001.docx]

**Table 1 supplementary: univariate analysis for OS1 and PFS1 by Cox proportional hazards model**

|  | **OS1** |  |  | **PFS1** |  |  |
| --- | --- | --- | --- | --- | --- | --- |
|  |  |  |  |  |  |  |
| **Characteristics** | **HR** | **95% CI** | **p-value** | **HR** | **95% CI** | **p-value** |
| **Outcome** |  |  |  |  |  |  |
| **NRR** |  |  |  |  |  |  |
| Relapsed | 17.7 | 9.3-33.4 | **0.000** | 64.0 | 31.9-128.3 | **0.000** |
| Refractory | 47.4 | 25.9-86.7 | **0.000** | 15,2 | 1.8-126.4 | **0.000** |
| **Age class** |  |  |  |  |  |  |
| < 60 y |  |  |  |  |  |  |
| 60-80 y | 1.2 | 0.8-2.0 | 0.379 | 1.4 | 0.9-2.1 | 0.135 |
| > 80 y | 5.7 | 3.3- 9.5 | **0.000** | 4.5 | 2.8-7.3 | **0.000** |
| **Sex** |  |  |  |  |  |  |
| Female |  |  |  |  |  |  |
| Male | 0.9 | 0.7-1.4 | 0.795 | 1.2 | 0.8- 1.7 | 0.299 |
| **Hb** |  |  |  |  |  |  |
| ≤10 g/dL |  |  |  |  |  |  |
| >10 g/dL | 0.6 | 0.4-1.0 | 0.06 | 0.6 | 0.4- 1.0 | **0.015** |
| **COO Hans** |  |  |  |  |  |  |
| GCB |  |  |  |  |  |  |
| Non GCB | 1.5 | 0.9-2.6 | 0.091 | 1.1 | 0.7-1.7 | 0.652 |
| **ECOG** |  |  |  |  |  |  |
| 0-1 |  |  |  |  |  |  |
| 2-4 | 1.4 | 0.9-2.2 | 0.098 | 1.2 | 0.7-1.8 | 0.495 |
| **Stage** |  |  |  |  |  |  |
| 1-2 |  |  |  |  |  |  |
| 3-4 | 1.9 | 1.1- 3.0 | **0.013** | 2.1 | 1.3- 3.3 | **0.002** |
| **Extranodal disease** |  |  |  |  |  |  |
| 0-1 |  |  |  |  |  |  |
| ≥2 | 1.6 | 1.1-2.3 | **0.009** | 1.6 | 1.1-2.2 | **0.006** |
| **Ki-67** |  |  |  |  |  |  |
| ≤70% |  |  |  |  |  |  |
| >70% | 1.0 | 0.7-1.5 | 0.969 | 0.9 | 0.6-1.3 | 0.516 |
| **LDH** |  |  |  |  |  |  |
| 0 |  |  |  |  |  |  |
| 1 | 2.6 | 1.6- 4.0 | **0.000** | 2.4 | 1.6-3.6 | **0.000** |
| **Type of treatment** |  |  |  |  |  |  |
| Anthracycline |  |  |  |  |  |  |
| High doses | 2.7 | 1.4- 5.1 | **0.003** | 2.0 | 1.1- 3.8 | **0.022** |
| Palliative | 3.9 | 2.1-7.4 | **0.000** | 3.0 | 1.7-5.6 | **0.000** |

**Table 2 supplementary: multivariate analysis for POD12 by logistic regression**

| \| **POD12** \| \| \| \| \| --- \| --- \| --- \| --- \| \|  \|  \|  \|  \| \| **Characteristics** \| **OR** \| **95% CI** \| **p-value** \| \| **(Intercept)** \| 0.01 \| 0.0-0.0 \| **<0.001** \| \| **Age class** \|  \|  \|  \| \| < 60 y \|  \|  \|  \| \| 60-80 y \| 2.9 \| 0.9-10.8 \| 0.076 \| \| > 80 y \| 8.8 \| 2.0-43.8 \| **0.005** \| \| **Hb** \|  \|  \|  \| \| ≤10 g/dL \|  \|  \|  \| \| >10 g/dL \| 0.8 \| 0.3-2.4 \| 0.690 \| \| **COO Hans** \|  \|  \|  \| \| GCB \|  \|  \|  \| \| Non GCB \| 1.0 \| 0.5-2.4 \| 0.856 \| \| **Sex** \|  \|  \|  \| \| Female \|  \|  \|  \| \| Male \| 1.4 \| 0.6-3.1 \| 0.451 \| \| **ECOG** \|  \|  \|  \| \| 0-1 \|  \|  \|  \| \| 2-4 \| 1.4 \| 0.5- 4.0 \| 0.500 \| \| **Stage** \|  \|  \|  \| \| 1-2 \|  \|  \|  \| \| 3-4 \| 7.7 \| 2-0-53.1 \| **0.011** \| \| **Extranodal disease** \|  \|  \|  \| \| 0-1 \|  \|  \|  \| \| ≥2 \| 1.1 \| 0.5-2.5 \| 0.857 \| \| **LDH** \|  \|  \|  \| \| 0 \|  \|  \|  \| \| 1 \| 2.4 \| 0.9-6.7 \| 0.080 \| \| **Ki-67** \|  \|  \|  \| \| ≤70% \|  \|  \|  \| \| >70% \| 1.0 \| 0.5-2.3 \| 0.950 \| \| **Type of treatment** \|  \|  \|  \| \| Anthracycline \|  \|  \|  \| \| High doses \| 1.8 \| 0.1, 15.7 \| 0.630 \| \| Palliative \| 2.1 \| 0.4-13.0 \| 0.392 \| |
| --- | --- | --- | --- | --- | --- | --- | --- | --- | --- | --- | --- | --- | --- | --- | --- | --- | --- | --- | --- | --- | --- | --- | --- | --- | --- | --- | --- | --- | --- | --- | --- | --- | --- | --- | --- | --- | --- | --- | --- | --- | --- | --- | --- | --- | --- | --- | --- | --- | --- | --- | --- | --- | --- | --- | --- | --- | --- | --- | --- | --- | --- | --- | --- | --- | --- | --- | --- | --- | --- | --- | --- | --- | --- | --- | --- | --- | --- | --- | --- | --- | --- | --- | --- | --- | --- | --- | --- | --- | --- | --- | --- | --- | --- | --- | --- | --- | --- | --- | --- | --- | --- | --- | --- | --- | --- | --- | --- | --- | --- | --- | --- | --- | --- | --- | --- | --- | --- | --- | --- | --- | --- | --- | --- | --- | --- | --- | --- | --- | --- | --- | --- | --- | --- | --- | --- | --- | --- | --- | --- | --- | --- | --- | --- | --- |

Abbreviations: POD12= progression/relapse/disease-related death within 12 months; OR= odds ratio; Hb= hemoglobin; COO= cell of origin; GCB= germinal centre B-cell; ECOG_PS = Eastern Cooperative Oncology Performance Status; LDH= lactate dehydrogenase
